# Income-related inequalities and decomposition of outpatient healthcare utilization in Saudi Arabia

**DOI:** 10.3389/fpubh.2026.1924371

**Published:** 2026-09-08

**Authors:** Khaled Shaeel Althabaiti, Monica Hunsberger, Sayem Ahmed, Jahangir Khan

**Affiliations:** 1 School of Public Health and Community Medicine, Institute of Medicine, Sahlgrenska Academy, University of Gothenburg, Gothenburg, Sweden; 2 Basic Nursing Sciences Department, College of Nursing, Taif University, Taif, Saudi Arabia; 3 Health Economics Research Group (HERG), Department of Health Sciences, Brunel University London, Uxbridge, London, United Kingdom; 4 Department of International Public Health, Liverpool School of Tropical Medicine, Liverpool, United Kingdom; 5 Department of Learning, Informatics, Management and Ethics, Karolinska Institutet, Stockholm, Sweden

**Keywords:** concentration index, private health insurance, Saudi Arabia, universal health coverage, vision 2030

## Abstract

**Background:**

The Kingdom of Saudi Arabia (KSA) has a dual health coverage system comprising government health coverage (GHC) and private health insurance (PHI). Evidence remains limited on how coverage type and socioeconomic status are associated with outpatient utilization, how utilization is distributed across income groups, and the extent to which income-related inequality is associated with need and non-need factors of healthcare utilization.

**Objective:**

To estimate and decompose income-related inequality in outpatient healthcare utilization in KSA by health coverage type and need and non-need factors of healthcare utilization.

**Methods:**

We analyzed data from the 2018 Saudi Family Health Survey, a nationally representative sample of 8,057 adults aged ≥19 years. Outpatient service utilization in the past 12 months was categorized as a binary variable (0 = no, 1 = yes). Monthly household income was used as the socioeconomic ranking variable in the inequality and decomposition analyses. Income-related inequality was measured using the Erreygers Concentration Index (ECI). A decomposition analysis was then performed to estimate the contributions of need and non-need factors, classified using Andersen’s Behavioral Model. These factors included health coverage types categorized as GHC or PHI, demographic, and health-related factors.

**Results:**

Outpatient utilization showed a significant overall pro-rich inequality (ECI = 0.122, *p* < 0.001). Inequality was greater within the PHI group (ECI = 0.257, *p* < 0.001) than within the GHC group (ECI = 0.040, *p* < 0.001). Decomposition analysis showed that Saudi nationality was the largest positive contributor to overall inequality (50.53%), followed by primary education (7.55%), female sex (4.65%), and unmarried status (3.01%). In contrast, residence in the Western region (−7.40%), chronic disease (−4.65%), mediocre self-rated health (−3.86%), and good self-rated health (−2.73%) reduced pro-rich inequality. PHI contributed negatively (−0.92%) to the overall inequality.

**Conclusion:**

Outpatient utilization in KSA showed significant pro-rich income-related inequality. This inequality was more pronounced within PHI than within GHC, particularly among non-Saudis and those with primary and intermediate education. Saudi nationality was the largest positive contributor, while PHI made a small negative contribution to overall inequality. These findings highlight the importance of evaluating Vision 2030 health coverage reforms not only by coverage expansion, but also by whether they reduce inequalities in outpatient healthcare utilization across income groups, supporting progress toward UHC.

## 1 Introduction

A key goal of global health is to ensure equitable access to quality health services for all, which is fundamental to achieving Universal Health Coverage (UHC). UHC means ensuring that everyone can access the quality health services they need without financial hardship (1). Achieving UHC, therefore, requires reducing inequalities in the utilization of healthcare services, as persistent disparities challenge its central goal of ensuring care for all (2). Health inequalities are systematic differences in healthcare services between populations that are often unjust and avoidable. These differences often reflect underlying social and economic disparities in the distribution of care among individuals with comparable healthcare needs (3).

Within the broader health-sector reforms initiated under Vision 2030, which started in 2016, the expansion of private-sector participation has increased the importance of private health insurance (PHI) alongside government health coverage (GHC) (4, 5). In KSA, GHC generally provides publicly financed healthcare services to Saudi citizens and eligible non-Saudi government-sector employees, whereas PHI is mainly linked to employer-sponsored coverage in the private sector (6). PHI in KSA is regulated through the Cooperative Health Insurance framework, which was established in 1999 to govern employer-sponsored private coverage (5). Employer-sponsored PHI became mandatory for expatriate workers, while its full extension to Saudi private-sector employees occurred in 2019 (4, 5). Therefore, the present study captures the pre-extension period and provides evidence on income-related inequality before this later phase of PHI expansion. This evolving dual coverage arrangement, publicly financed access through GHC and employer-sponsored access through PHI, raises important questions about whether different coverage routes are associated with socioeconomic inequalities in healthcare utilization.

Differences in the design and access conditions of GHC and PHI may be associated with different utilization patterns (7, 8). Access under GHC may be limited by public-sector capacity and workforce limitations, which may be associated with long waiting times and delayed care (9). Meanwhile, PHI coverage may vary by occupation and income group, with higher-income workers generally receiving broader benefits, whereas lower-income expatriates are often limited to basic packages with restricted provider networks, copayments, and limits on covered services (10–12). These mechanisms may contribute to disparities in financial protection, service availability, continuity of care, and out- and inpatient utilization, raising critical equity concerns in light of Vision 2030 reforms that aim to expand coverage, enhance efficiency, and ensure equitable access to healthcare services (10–12).

Previous research in KSA found that the uptake of preventive health checkups was positively associated with higher income and better education (13). The same study also showed that utilization was disproportionately concentrated among higher-income groups (i.e., a pro-rich distribution), and decomposition analysis demonstrated that income (24.4%), region (23.4%), and education (17.8%) contributed the largest share of the observed overall inequality (13). However, that study focused primarily on preventive services, such as health checkups, leaving open the question of whether similar socioeconomic and coverage-related inequalities exist in out- and inpatient healthcare utilization more broadly. Outpatient utilization extends beyond preventive check-ups to include routine clinical care, chronic disease follow-up, and key access pathways, such as referrals and continuity of care.

A recent study in Iran has analyzed healthcare utilization inequality using the ECI and found that pro-rich inequality in outpatient treatment persisted from 2008 to 2016, with income, education, wealth, and PHI being the main contributing factors (14). In KSA, most empirical studies on equity in healthcare utilization have focused primarily on out-of-pocket payments for healthcare (6) or preventive services, such as health check-ups, and have reported that healthcare expenditures and utilization vary considerably across socioeconomic groups (income groups, educational levels, and regions) (13).

In KSA, prior studies have shown very little evidence of inequalities in healthcare utilization. A recent systematic review of the PHI system found that most studies focused on financial protection and access to care, rather than on income-related inequality in outpatient healthcare utilization across health coverage types (5). By contrast, international research from Iran (14) and China (15) have reported socioeconomic inequalities in outpatient and broader healthcare utilization. This is consistent with existing evidence suggesting that outpatient healthcare services are used more frequently by individuals of higher socioeconomic status.

Under the Vision 2030 agenda, KSA has been moving toward a more mixed health coverage system in which both GHC and PHI play central roles. This ongoing policy direction and the dual coverage arrangement may be associated with outpatient healthcare utilization and raise concerns about income-related inequalities between the two coverage types (4). Studying income-related inequality in outpatient healthcare utilization is particularly relevant in KSA, given its major health-sector reforms aimed at improving efficiency and ensuring sustainability (4). However, to our knowledge, this is the first study to quantify and decompose income-related inequality in outpatient utilization by health coverage type in KSA, providing important pre-extension evidence for monitoring equity under Vision 2030.

### 1.1 Rationale

The full extension of employer-sponsored PHI to Saudi private-sector workers in 2019 represented an important first-phase reform milestone under Vision 2030. Examining income-related inequality in outpatient healthcare utilization before this extension provides important pre-extension evidence for monitoring equity in the post-extension period. Outpatient care is important to study because it is often the first and most frequent point of contact with the health system. Compared with inpatient care, outpatient utilization may be more sensitive to differences in insurance coverage, affordability, access, appointment availability, and care-seeking behavior (16). According to Andersen’s Behavioral Model, outpatient utilization should primarily reflect healthcare need rather than non-need factors. However, pro-rich income-related inequality may be expected in the Saudi context because coverage type, nationality, education, and region may be associated with access to outpatient services independently of healthcare need. Therefore, decomposing income-related inequality into need and non-need contributors helps determine whether the observed inequality is mainly associated with healthcare need or with non-need factors that may suggest inequity.

### 1.2 Aim of the study

To estimate and decompose income-related inequality in outpatient healthcare utilization in KSA by health coverage type, and need and non-need factors of healthcare utilization.

### 1.3 Research questions

1. What is the extent of income-related inequality in outpatient healthcare utilization in KSA?
2. How does the extent of income-related inequality in outpatient healthcare utilization differ between individuals with PHI and GHC?
3. Which demographic, regional, health-related, and coverage-related factors contribute most to income-related overall inequality in outpatient healthcare utilization, and what is the specific contribution of PHI?

## 2 Materials and methods

### 2.1 Data source and sample

This study used a cross-sectional design based on data from the 2018 Saudi Family Health Survey (SFHS). A total of 12,876 individuals participated in the survey. For the present analysis, the sample was restricted to adults aged ≥19 years, resulting in an analytical sample of 8,057 participants. The ≥19-year threshold was prespecified to approximate the transition beyond secondary education into post-secondary study or labor-market participation in the Saudi context. The SFHS used a two-stage stratified cluster sampling design covering all 13 administrative regions of KSA. In the first stage, enumeration areas, representing primary sampling units, were selected within strata with probability proportional to size. In the second stage, households were selected within each selected area using systematic random sampling. Monthly household income was not used as a stratification or household-selection criterion and was collected subsequently during the survey interview. The microdata was obtained from the General Authority for Statistics (GASTAT) in an analysis-ready format. According to GASTAT, the analytical file provided for this study was self-weighted; therefore, no additional sampling weights or post-stratification adjustments were applied by the authors. However, separate survey design identifiers, such as strata and primary sampling units, were not included in the released dataset. The 2018 SFHS wave was conducted before the 2019 policy expansion of employer-mandated PHI. At that time, employer-sponsored PHI mainly applied to expatriate workers in the private sector, although some Saudi private-sector employees were already covered by employer-sponsored PHI before 2019 (5, 6, 17).

### 2.2 Variables used

#### 2.2.1 Outcome variable

Outpatient healthcare utilization was self-reported by individual survey participants and defined as any outpatient visit in the past 12 months (yes/no), reflecting realized service use rather than direct measures of access or unmet need. Consistent with the study’s focus on whether individuals used outpatient services, utilization was operationalized as a binary indicator (any visit vs. no visit) rather than visit frequency.

#### 2.2.2 Ranking variable

Monthly household income was used as reported to rank individuals from lowest to highest income. Household size information was not available in the released analytical dataset; therefore, household income could not be equivalized. This may introduce measurement error in the socioeconomic ranking and may affect the inequality estimates. For the inequality analysis, monthly household income categories were ordered from lowest to highest income (<5,000 SAR to ≥20,000 SAR) and coded as an ordinal rank variable. This income rank was used as the socioeconomic ranking variable for estimating the ECI (13, 18). Income was used as the ranking variable since it is one of the most appropriate indicators of the socioeconomic condition of individuals or households (19).

#### 2.2.3 Decomposing factors (predisposing, enabling, need)

Decomposing factors were organized into need and non-need categories, drawing on Andersen’s Behavioral Model of Health Services Use (BMHSU) as a classification guide and in line with previous inequality studies (14, 15, 20). Non-need factors comprised predisposing and enabling characteristics. Predisposing factors included sex (male, female), marital status (married, unmarried), nationality (Saudi, non-Saudi), and education (illiterate, primary, intermediate, secondary, higher education). Enabling factors included the type of health coverage, which was self-reported in the SFHS and coded as GHC (including Saudi citizens and non-Saudi public-sector employees) or PHI (including employer-sponsored and individual plans), and region of residence, covering all five broad regions: Central, East, North, South, and West (21). Need proxies included age (19–29, 30–39, 40–49, 50–59, ≥60), self-rated health status (very good, good, mediocre, poor), and the presence of chronic disease (yes, no). Age was treated as a proxy for need because morbidity and functional limitations generally increase with age, even though age itself is not a direct measure of morbidity. In the absence of a direct measure of healthcare need in the SFHS, age, self-rated health status, and chronic disease were used as proxies for need, consistent with prior inequality studies (14, 15, 22). In the Saudi context, these need proxies may under-capture need among expatriates (e.g., healthy-worker selection and immigration-related health screening), which may affect the interpretation of horizontal inequity (23).

### 2.3 Data analysis

#### 2.3.1 Descriptive statistics

Descriptive statistics were used to summarize the demographic, socioeconomic, and health-related characteristics of the study population. Frequencies and percentages were calculated for all categorical variables to describe the characteristics of the study population.

#### 2.3.2 Erreygers concentration index

Income-related inequality in outpatient utilization was quantified using the ECI (24). The standard concentration index can be difficult to interpret for bounded outcomes because its bounds depend on the mean when the outcome is binary; the Erreygers correction provides a normalized index for a 0–1 variable. Therefore, ECI is appropriate for binary outpatient utilization and facilitates comparisons of income-related inequality across subgroups. In addition, the ECI captures both the direction of inequality (pro-rich or pro-poor) and its magnitude when applied to bounded health variables (24, 25). In this study, the ECI was calculated for the main outcome variable, which is outpatient healthcare utilization (24, 25).

The ECI was estimated directly from individual-level data in Stata using the conindex command with the Erreygers correction. Individuals were ranked according to ordered monthly household income categories. Because income was reported in categories rather than exact values, no income midpoints were used; the conindex command generated the corresponding fractional ranks and accounted for tied income categories. In addition to the overall estimate, subgroup-specific ECIs were computed for the subgroups presented in Table 1; within each subgroup, individuals were ranked by income, and the ECI was estimated separately. Thus, subgroup ECIs reflect income-related inequality in outpatient utilization within each subgroup rather than differences between subgroups. For bounded outcomes, the ECI can be expressed equivalently as (24, 26):


ECIγ=4×μ×Cγ

**Table 1:** Erreygers concentration index for outpatient healthcare utilization across income categories, by health coverage type and sociodemographic characteristics.

Variables		Proportions represent the share of individuals utilizing outpatient healthcare services across income groups for each subgroup					Concentration index	
		Lowest income group (< 5,000 SAR)	2nd income group (5,000–9,999 SAR)	3rd income group (10,000–14,999SAR)	4th income group (15,000–19,999 SAR)	Highest income group (≥20,000 SAR)	Erreygers Concentration Index (95% CIs)	*p* value
Healthcare utilization	Utilized outpatient healthcare services	(*n* = 2,527) 0.654	(*n* = 2,823) 0.775	(*n* = 1,385) 0.789	(*n* = 652) 0.760	(*n* = 670) 0.828	0.122(0.101, 0.143)	<0.001
Type of health coverage	GHC	(*n* = 1,594) 0.747	(*n* = 2,199) 0.804	(*n* = 1,114) 0.799	(*n* = 493) 0.764	(*n* = 522) 0.825	0.040 (0.017,0.064)	<0.001
	PHI	(*n* = 933) 0.495	(*n* = 624) 0.673	(*n* = 271) 0.749	(*n* = 159) 0.748	(*n* = 148) 0.837	0.257 (0.214, 0.301)	<0.001
Nationality	Non-Saudi	(*n* = 1,113) 0.466	(*n* = 478) 0.552	(*n* = 136) 0.764	(*n* = 67) 0.567	(*n* = 86) 0.799	0.170 (0.126, 0.216)	<0.001
	Saudi	(*n* = 1,394) 0.807	(*n* = 2,345) 0.820	(*n* = 1,249) 0.792	(*n* = 585) 0.802	(*n* = 584) 0.835	−0.005 (−0.017, 0.027)	0.643
Sex	Male	(*n* = 1,550) 0.571	(*n* = 1,519) 0.751	(*n* = 695) 0.782	(*n* = 328) 0.740	(*n* = 342) 0.830	0.195 (0.166, 0.225)	<0.001
	Female	(*n* = 977) 0.786	(*n* = 1,304) 0.802	(*n* = 690) 0.795	(*n* = 324) 0.780	(*n* = 328) 0.826	0.012 (−0.016, 0.042)	0.395
Age	19–29	(*n* = 777) 0.638	(*n* = 920) 0.734	(*n* = 437) 0.773	(*n* = 213) 0.751	(*n* = 236) 0.817	0.125 (0.087, 0.163)	<0.001
	30–39	(*n* = 620) 0.577	(*n* = 724) 0.754	(*n* = 417) 0.767	(*n* = 152) 0.743	(*n* = 127) 0.874	0.180 (0.138, 0.224)	<0.001
	40–49	(*n* = 419) 0.544	(*n* = 427) 0.754	(*n* = 219) 0.803	(*n* = 143) 0.797	(*n* = 128) 0.773	0.214 (0.161, 0.268)	<0.001
	50–59	(*n* = 303) 0.722	(*n* = 355) 0.805	(*n* = 144) 0.777	(*n* = 75) 0.760	(*n* = 105) 0.790	0.047 (−0.011, 0.105)	0.113
	≥60	(*n* = 408) 0.862	(*n* = 397) 0.904	(*n* = 168) 0.881	(*n* = 69) 0.753	(*n* = 74) 0.932	0.007 (−0.035, 0.049)	0.739
Education level	Illiterate	(*n* = 268) 0.858	(*n* = 134) 0.932	(*n* = 42) 0.881	(*n* = 15) 1.000	(*n* = 15) 0.933	0.066 (0.009, 0.125)	0.025
	Primary school	(*n* = 802) 0.632	(*n* = 540) 0.800	(*n* = 171) 0.888	(*n* = 82) 0.743	(*n* = 76) 0.568	0.195 (0.151,0.240)	<0.001
	Intermediate school	(*n* = 364) 0.560	(*n* = 356) 0.803	(*n* = 117) 0.820	(*n* = 43) 0.767	(*n* = 41) 0.878	0.245 (0.184,0.306)	<0.001
	Secondary school	(*n* = 695) 0.680	(*n* = 999) 0.743	(*n* = 484) 0.735	(*n* = 226) 0.747	(*n* = 203) 0.827	0.071 (0.034,0.109)	0.002
	Higher education	(*n* = 398) 0.600	(*n* = 794) 0.759	(*n* = 571) 0.793	(*n* = 286) 0.762	(*n* = 335) 0.809	0.120 (0.082,0.160)	<0.001
Marital status	Married	(*n* = 1,713) 0.634	(*n* = 1,953) 0.764	(*n* = 923) 0.766	(*n* = 401) 0.778	(*n* = 369) 0.829	0.092 (0.058, 0.128)	<0.001
	Unmarried	(*n* = 814) 0.695	(*n* = 870) 0.801	(*n* = 462) 0.837	(*n* = 251) 0.733	(*n* = 301) 0.827	0.133 (0.107, 0.160)	<0.001
Chronic diseases	No	(*n* = 1,799) 0.567	(*n* = 2,050) 0.735	(*n* = 1,043) 0.756	(*n* = 517) 0.742	(*n* = 526) 0.806	0.174 (0.149,0.200)	<0.001
	Yes	(*n* = 728) 0.869	(*n* = 773) 0.882	(*n* = 342) 0.891	(*n* = 135) 0.829	(*n* = 144) 0.909	0.011 (−0.019,0.042)	0.470
Self-rated health status	Very Good	(*n* = 1,676) 0.559	(*n* = 1,993) 0.727	(*n* = 1,050) 0.752	(*n* = 509) 0.725	(*n* = 509) 0.805	0.171 (0.146, 0.198)	<0.001
	Good	(*n* = 493) 0.780	(*n* = 560) 0.878	(*n* = 216) 0.902	(*n* = 104) 0.894	(*n* = 112) 0.866	0.094 (0.054, 0.134)	<0.001
	Mediocre	(*n* = 265) 0.901	(*n* = 227) 0.898	(*n* = 93) 0.892	(*n* = 27) 0.814	(*n* = 29) 0.965	−0.006 (−0.044, 0.056)	0.808
	Poor	(*n* = 93) 0.978	(*n* = 43) 1.000	(*n* = 26) 1.000	(*n* = 12) 1.000	(*n* = 20) 1.000	0.021 (−0.009, 0.052)	0.172
Regions	Center (Riyadh, Qassim)	(*n* = 456) 0.526	(*n* = 676) 0.693	(*n* = 294) 0.642	(*n* = 171) 0.590	(*n* = 110) 0.863	0.122 (0.073, 0.173)	<0.001
	East (Eastern Province)	(*n* = 360) 0.716	(*n* = 331) 0.800	(*n* = 251) 0.768	(*n* = 105) 0.819	(*n* = 129) 0.759	0.049 (−0.005, 0.104)	0.074
	North (Northern Borders, Al-Jouf, Hail)	(*n* = 382) 0.604	(*n* = 408) 0.742	(*n* = 225) 0.875	(*n* = 83) 0.759	(*n* = 166) 0.861	0.213 (0.161, 0.266)	<0.001
	Southern (Baha, Jizan, Asir, Najran)	(*n* = 536) 0.735	(*n* = 561) 0.836	(*n* = 262) 0.855	(*n* = 154) 0.922	(*n* = 146) 0.815	0.110 (0.069, 0.152)	<0.001
	West (Makkah, Madinah, Tabuk)	(*n* = 793) 0.668	(*n* = 847) 0.806	(*n* = 353) 0.824	(*n* = 139) 0.748	(*n* = 119) 0.840	0.127 (0.089, 0.166)	<0.001

Where μ is the mean of the outcome in the relevant subgroup and C_y_ is the standard concentration index of the outcome (24, 26). No income midpoints were used because income was reported in categories. 95% CIs were calculated using the Wald formula (ُEECI ± 1.96 × SE), where SE is the standard error from the ECI estimation (27).

#### 2.3.3 Decomposition

The decomposition identifies how much each factor contributes to the observed income-related inequality (28). To decompose overall inequality in outpatient healthcare utilization, we estimated a binary logistic regression model with outpatient healthcare utilization (1 = yes, 0 = no) as the dependent variable and the selected explanatory variables as predictors. Because the outcome is binary and logit coefficients are expressed on the log-odds scale, we derived average marginal effects (AMEs) to quantify covariate effects on the probability scale. Accordingly, the decomposition uses these marginal effects interpreted as the average change in the predicted probability of outpatient utilization, rather than log-odds coefficients, and these AMEs were then used to compute elasticities within the decomposition framework (29, 30).

Monthly household income was used as the socioeconomic ranking variable for estimating income-related inequality and was therefore not included as an explanatory variable in the decomposition model. The decomposition aimed to identify how need and non-need factors contributed to inequality across the income distribution; including income itself as an explanatory variable would risk explaining income-related inequality by the same variable used to define the socioeconomic ranking.

The decomposition analysis was conducted in Stata using logistic regression (logit), followed by margins, dydx(*) (where * refers to all explanatory variables in the model) to obtain average marginal effects. The concentration index of each determinant (Cj) was estimated using conindex determinant, rankvar(monthly household income) truezero. The resulting marginal effects and concentration indices were then used to calculate elasticities and the absolute and relative contributions according to the equations described below.

For each explanatory variable, elasticity was computed as (28, 31):


$$\text{Elasticit}y_{j}=\frac{\left(\mathrm{AM}E_{j}\times {\bar{X}}_{j}\right)}{\mu }$$

Where $\text{Elasticit}y_{j}$ represents the elasticity of outpatient utilization with respect to determinant X_j_. (AME_j_) is the average marginal effect from the logistic regression model on the probability scale (for binary indicators, this is the discrete change in predicted probability when X_j_ changes from 0 to 1 relative to the reference group); (X̄_j_) is the mean of the explanatory variable (e.g., type of health coverage, nationality, education level, and age group), and (μ) is the mean of the outcome variable, which is outpatient healthcare utilization (28, 31). For categorical variables, each category was included as an indicator (dummy) variable relative to a reference group, and elasticities were computed for each indicator. In line with previous studies, elasticity was used as the weight of each explanatory variable in the decomposition, reflecting its contribution to overall inequality (30).

The absolute contribution of each determinant to overall inequality on the Erreygers scale was calculated as (24, 28, 32):


$$\text{Contribution}=\text{Elasticit}y_{j}\times 4\times \mu \times Cj$$

Where C_j_ is the concentration index of determinant X_j_, indicating how each explanatory variable is distributed across the income ranking. C_j_ was calculated using the same monthly household income ranking variable. A positive C_j_ means that the determinant is more concentrated among higher-income individuals, whereas a negative C_j_ means that the determinant is more concentrated among lower-income individuals. The overall inequality in outpatient utilization is summarized by ECI_y_. These decomposition contributions are descriptive and reflect associations combined with the income-related distribution of each determinant. The relative contribution was computed by dividing each contribution by the overall ECI_y_ and expressing it as a percentage. Positive contributions reflect an increase in inequality, whereas negative contributions indicate a reduction in such inequality (26, 28).


Relative Contribution=(Contribution÷OverallECI)×100)

Multicollinearity was assessed using the generalized variance inflation factor (GVIF), and all values were below 5, indicating no serious multicollinearity among the explanatory variables. All statistical analyses were performed using Stata/SE version 18.0, which was employed for data preparation, descriptive analysis, regression modeling, and inequality measurement and decomposition.

#### 2.3.4 Ethical clearance

This study used secondary, anonymized data from the SFHS, which was conducted, commissioned, funded, and managed in 2018 by the GASTAT. We obtained official permission from GASTAT to access and use the dataset, which was provided in fully anonymized form with no personal identifiers. As the dataset was secondary and fully anonymized, additional ethical approval in KSA was not applicable. Ethical approval for the present analysis was obtained from the Swedish Ethical Review Authority (Registration number: 2025–03265-01).

## 3 Results

Table 2. summarizes the characteristics of the 8,057 adults included in the 2018 SFHS. A total of 74.3% of respondents reported using outpatient healthcare services during the 12 months preceding the survey. Outpatient healthcare utilization reflects any outpatient contact in the past 12 months (including preventive and minor visits). Health coverage was primarily GHC (73.5%), and 26.5% had PHI. Regarding household income, most participants (66.5%) were in the lowest income category (<10,000 SAR), whereas only 8.3% were in the highest income category (≥20,000 SAR). More than half were male (55.1%), and the majority were in the younger age group (19–29 years) (32.1%). Almost one-third (32.4%) had completed secondary education, followed by those with higher education (university qualification or postgraduate degree) (29.6%). Most respondents were married (66.5%), and 76.4% were Saudis.

**Table 2:** Characteristics of the study population (*n* = 8,057).

Characteristics		Family health survey 2018	
		*N* (8,057)	%
Have you utilized outpatient healthcare services	Yes	5,987	74.3%
	No	2,070	25.7%
Type of health coverage	GHC	5,922	73.5%
	PHI	2,135	26.5%
Monthly income categories	Lowest: < 5,000 SAR	2,527	31.4%
	2nd: 5,000–9,999 SAR	2,823	35.1%
	3rd: 10,000–14,999 SAR	1,385	17.1%
	4th: 15,000–19,999 SAR	652	8.1%
	Highest: ≥20,000 SAR	670	8.3%
Sex	Male	4,434	55.1%
	Female	3,623	44.9%
Age	19–29	2,583	32.1%
	30–39	2,040	25.3%
	40–49	1,336	16.6%
	50–59	982	12.2%
	≥60	1,116	13.8%
Education level	Illiterate	474	5.9%
	Primary school	1,671	20.7%
	Intermediate school	921	11.4%
	Secondary school	2,607	32.4%
	Higher education	2,384	29.6%
Marital status	Married	5,359	66.5%
	Unmarried	2,698	33.5%
Nationality	Saudi	6,157	76.4%
	Non-Saudi	1,900	23.6%
Chronic diseases	Yes	2,122	26.3%
	No	5,935	73.7%
Self-rated health status	Very Good	5,737	71.2%
	Good	1,485	18.5%
	Mediocre	641	7.9%
	Poor	194	2.4%
Regions	Center (Riyadh, Qassim)	1,707	21.2%
	East (Eastern Province)	1,176	14.6%
	North (Northern Borders, Al-Jouf, Hail)	1,264	15.7%
	South (Baha, Jizan, Asir, Najran)	1,659	20.6%
	West (Makkah, Madinah, Tabuk)	2,251	27.9%

Chronic conditions were reported by 26.3% of the respondents. Most participants (71.2%) rated their health as very good, and only 7.9 and 2.4% reported mediocre or poor health, respectively. Geographically, participants were representative of the five main administrative regions of KSA, who were mostly concentrated in the Western region (Makkah, Madinah, Tabuk) (27.9%), and the Central region (Riyadh, Qassim) 21.2%.

In Table 1, the ECI of outpatient healthcare utilization of all individuals was 0.122 (95% CI: 0.101–0.143, *p* < 0.001), indicating a statistically significant pro-rich inequality. Subgroup-specific ECIs further highlighted that inequality was considerably higher among individuals with PHI (ECI = 0.257, 95% CI: 0.214–0.301, *p* < 0.001) than among those with GHC (ECI = 0.040, 95% CI: 0.017–0.064, *p* < 0.001). Non-Saudi participants also demonstrated a substantial pro-rich inequality (ECI = 0.170, 95% CI: 0.126–0.216, *p* < 0.001). Gender-based differences were evident, with males showing higher inequality (ECI = 0.195, 95% CI: 0.166–0.225, *p* < 0.001).

The highest inequality was observed among those aged 40–49 (ECI = 0.214, 95% CI: 0.161–0.268, *p* < 0.001). Regarding education level, the most pronounced inequality was observed among individuals with intermediate education (ECI = 0.245, 95% CI: 0.184–0.306, *p* < 0.001). Unmarried individuals showed a significantly high positive ECI (0.133, 95% CI: 0.107–0.160, *p* < 0.001). Participants without chronic conditions (ECI = 0.174, 95% CI: 0.149–0.200, *p* < 0.001), and individuals reporting “very good” health (ECI = 0.171, 95% CI: 0.146–0.198, *p* < 0.001) showed significant pro-rich inequality. The Northern region showed significantly high pro-rich inequality (ECI = 0.213, 95% CI: 0.161–0.266, *p* < 0.001).

Table 3 presents the decomposition results, showing the absolute and relative contribution of each explanatory variable to the observed overall pro-rich income-related inequality in outpatient healthcare utilization. Saudi nationality was the largest positive contributor to the overall inequality (50.53%). However, PHI had a very small negative contribution (−0.92%) to overall inequality. Other positive contributors, in addition to Saudi nationality, included primary education (7.55%), female sex (4.65%), unmarried status (3.01%), residence in the Eastern region (2.77%), residence in the Northern region (2.26%), higher education (1.24%), and intermediate education (0.73%). In contrast, several factors made negative contributions, indicating a reduction in the observed inequality. These included residence in the Western region (−7.40%), chronic disease (−4.65%), mediocre self-rated health (−3.86%), good self-rated health (−2.73%), poor self-rated health (−2.56%), older age (≥60 years; −1.11%), secondary education (−1.06%), and PHI (−0.92%). Overall, the included variables explained 48.64% of the total inequality, while 51.36% remained unexplained.

**Table 3:** Decomposition of income-related inequality in outpatient healthcare utilization.

Variables		Marginal effect	(X-)	(y-)	Elasticity	Cj	Absolute contribution	Relative share
Type of health Coverage	GHC (Ref)							
	PHI	0.0086	0.265	0.743	0.0031	−0.1233	−0.0011	−0.92%
Nationality	Non-Saudi (Ref)							
	Saudi	0.2100	0.764	0.743	0.2160	0.0959	0.0615	50.53%
Sex	Male (Ref)							
	Female	0.0600	0.450	0.743	0.0363	0.0524	0.0057	4.65%
Age	19–29 (Ref)							
	30–39	0.0285	0.253	0.743	0.0097	−0.0011	0.0000	−0.03%
	40–49	0.0103	0.134	0.743	0.0019	0.0279	0.0001	0.13%
	50–59	0.0158	0.122	0.743	0.0026	0.0089	0.0000	0.06%
	≥60	0.0319	0.139	0.743	0.0060	−0.0762	−0.0013	−1.11%
Education	Illiterate (Ref)							
	Primary school	−0.0537	0.207	0.743	−0.0150	−0.2068	0.0092	7.55%
	Intermediate school	−0.0139	0.114	0.743	−0.0021	−0.1404	0.0009	0.73%
	Secondary school	−0.0262	0.324	0.743	−0.0114	0.0379	−0.0013	−1.06%
	Higher education	0.0059	0.296	0.743	0.0024	0.2168	0.0015	1.24%
Marital status	Married (Ref)							
	Unmarried	0.0628	0.335	0.743	0.0283	0.0436	0.0037	3.01%
Chronic diseases	No (Ref)							
	Yes	0.1018	0.263	0.743	0.0360	−0.0529	−0.0057	−4.65%
Self-rated health status	Very Good (Ref)							
	Good	0.1083	0.184	0.743	0.0268	−0.0417	−0.0033	−2.73%
	Mediocre	0.1007	0.080	0.743	0.0108	−0.1459	−0.0047	−3.86%
	Poor	0.2580	0.024	0.743	0.0083	−0.1258	−0.0031	−2.56%
Regions	Central (Riyadh, Qassim) (Ref)							
	Eastern (Eastern Province)	0.0995	0.146	0.743	0.0196	0.0580	0.0034	2.77%
	Northern (Northern Borders, Al-Jouf, Hail)	0.1002	0.156	0.743	0.0210	0.0441	0.0028	2.26%
	Southern (Baha, Jizan, Asir, Najran)	0.1464	0.205	0.743	0.0404	0.0002	0.0000	0.01%
	Western (Makkah, Madinah, Tabuk)	0.1051	0.279	0.743	0.0395	−0.0768	−0.0090	−7.40%
Total explained inequality							0.0592	48.64%
Residual (unexplained)							0.0626	51.36%
Overall inequality (ECI_y_)							0.1218	100%

GHC, government health coverage; PHI, private health insurance; AME, average marginal effect; X̄, mean of the explanatory variable; Ӯ, mean outpatient utilization; Cj, concentration index of each determinant, indicating how that determinant is distributed across the income ranking.

This unexplained component should not be interpreted as a weakness of the decomposition model, but rather as reflecting the limited availability of access-related and insurance-related variables in the SFHS dataset. Specifically, it may reflect unmeasured or unavailable factors that vary by income and are relevant to outpatient use, such as service availability and provider capacity, waiting times, travel distance, benefit generosity, provider-network restrictions, and administrative barriers (33). Differences in health literacy, awareness of entitlements, and unmeasured health need may also contribute. Accordingly, the residual should be interpreted as an unexplained component rather than evidence of causal effects.

## 4 Discussion

This study indicates substantial pro-rich income-related inequality in outpatient healthcare utilization among individuals in KSA. Income-related inequality in outpatient healthcare utilization was markedly higher among PHI holders than among those covered by GHC. However, PHI made a very small negative contribution to the overall inequality. This difference reflects that the subgroup ECI and decomposition contribution measure different things. The high ECI among PHI holders indicates pro-rich inequality within the PHI group, whereas the decomposition contribution reflects the role of PHI status in explaining overall inequality in the full sample. Because PHI had a small marginal association with outpatient utilization and was relatively more concentrated among lower-income individuals, its contribution to overall inequality was small and negative.

These findings are consistent with the expectation that outpatient healthcare utilization in KSA may be shaped not only by healthcare need but also by non-need factors related to socioeconomic position and coverage arrangements. From an equity perspective, differences in utilization associated with need-related factors, such as age, chronic disease, and poorer self-rated health, may reflect differences in healthcare need rather than inequity. In contrast, differences associated with non-need factors, such as nationality, education, region, and health coverage type, may indicate inequity in realized outpatient service use. In this study, the decomposition results suggest that much of the explained income-related inequality was associated with non-need factors, particularly nationality, rather than the available need proxies. The higher pro-rich inequality observed among PHI holders is consistent with broader international evidence showing that healthcare utilization is often concentrated among higher-income groups (14, 15).

This PHI subgroup finding should be distinguished from the decomposition result for the PHI variable (Tables 1, 3). Although outpatient utilization within PHI holders was substantially pro-rich, PHI status made a small negative contribution to overall income-related inequality. PHI coverage itself was relatively more concentrated among lower-income individuals than higher-income individuals. Because PHI holders represented a relatively small proportion of the total sample, and because the marginal association between PHI and outpatient utilization was small, PHI did not materially contribute to the overall pro-rich inequality, although substantial income-related inequality existed within the PHI group. Therefore, the substantial inequality observed among PHI holders reflects a within-group pattern, whereas the small negative PHI contribution reflects its limited role in explaining overall inequality across the full study population.

In the decomposition of overall pro-rich income-related inequality in outpatient healthcare utilization, Saudi nationality was the largest contributor to the explained inequality. This finding may reflect the way nationality is linked to healthcare entitlement, employment sector, income position, and coverage arrangements in KSA (5, 6, 23, 34). Saudi citizens generally access publicly financed care through GHC, whereas many non-Saudis rely on employer-sponsored PHI, with coverage conditions that may vary by employers, occupations, and income levels (5, 6, 23). Because nationality is unevenly distributed across income groups and closely linked to coverage arrangements, it may represent an important pathway through which income-related inequality in outpatient utilization is observed (5, 6, 23). This interpretation aligns with prior studies in KSA showing that PHI premium levels and benefit generosity vary by employment characteristics, including job type, employer size; and job position, higher-income employees tend to have more comprehensive coverage than those in lower positions (5, 6, 23). Several need-related factors, including chronic disease, poorer self-rated health, and older age, made negative contributions, suggesting that the available need proxies reduced, rather than increased, the observed pro-rich inequality. Overall, these findings suggest that the explained component of income-related inequality was more closely associated with non-need factors, particularly nationality and coverage-related arrangements, than with the available need proxies (14, 15, 22, 35). However, healthcare need is only partially captured by the available proxies (age, self-rated health, and chronic disease condition) and may differ systematically across groups (e.g., due to expatriate screening and healthy-worker effects).

The finding of pro-rich income-related inequality is consistent with previous evidence from KSA and international studies from Iran and China. In KSA, previous research has shown socioeconomic inequalities in preventive health check-ups, particularly by income, education, and region. International studies from Iran and China have reported socioeconomic inequalities in outpatient and healthcare utilization. However, comparisons across countries should be interpreted cautiously because PHI systems differ substantially in their design and role. In KSA, PHI is largely employer-sponsored and linked to private-sector employment, whereas in other countries, PHI may operate as complementary, supplementary, or substitutive coverage (13, 14, 17, 36).

In 2018, before the full extension of employer-mandated PHI to Saudi private-sector workers, PHI was predominantly associated with expatriate and private-sector employment, which may be associated with the higher observed inequality among expatriates. Low-income expatriates were often limited to basic PHI packages with restricted provider networks, while higher-income expatriates tended to have access to more comprehensive plans that covered a wider range of benefits, including preventive services (5, 6). Alongside these income-related differences in PHI benefit packages, additional barriers, such as language difficulties and limited awareness of PHI coverage, may have further contributed to the observed disparities in healthcare utilization (8, 11, 37). Moreover, expatriate workers are required to undergo mandatory medical examinations as part of the work-visa process (pre-departure screening in the home country and a repeat examination upon arrival in KSA), which may partly explain why non-Saudis more often report good self-rated health (38). In addition, individuals in good health are more likely to hold higher-income positions that generally provide access to more comprehensive PHI benefit packages, which may be associated with more frequent outpatient service use, particularly preventive check-up services, and to access inpatient care when needed (11, 13, 23, 39). This may partly contribute to the observed pro-rich inequality. Evidence from France similarly shows that while GHC reduces disparities, complementary PHI is associated with greater utilization among higher-income households, consistent with the possibility that PHI may be associated with wider socioeconomic gaps in healthcare access (40). These results indicate that equity-oriented reforms should prioritize regulating PHI benefit packages to ensure a minimum essential level of coverage, particularly for low-income expatriates, to align with Vision 2030’s commitment to improving fairness and reducing inequities in access to care.

Income-related inequality was most pronounced among individuals aged 40–49 years. One possible explanation is that individuals aged 40–49 are commonly active in the labor market, where income level and employment conditions may influence access to employer-sponsored PHI and outpatient care. Therefore, the higher pro-rich inequality in this age group may reflect income-related differences in employment-linked coverage and access conditions rather than age alone (41). Individuals in lower-income groups may be more likely to report poorer health and to have less generous PHI coverage (e.g., more limited benefits or provider networks), which may be associated with lower outpatient service use, even among those with potentially higher need as captured by the available proxies in this study, namely age, chronic disease, and self-rated health (23, 42). Needs increase with individuals’ progress through midlife, particularly with the onset of chronic conditions (42). This pattern is similar to associations reported in other contexts, such as China and Iran, where midlife has been described as a potential turning point in widening health disparities (15, 42). These findings may help inform efforts to reduce income-related inequalities in access and utilization, particularly by promoting earlier preventive interventions, such as check-ups in the workplace for younger adults, to address disparities that appear in midlife in KSA.

Significant pro-rich inequality was observed among males. Men from different socioeconomic backgrounds prioritize health differently; higher-income men may view health as an investment in their long-term productivity and quality of life, as well as men who maintain high-income pathways enjoy substantially better physical and mental health at midlife than those with low incomes, partly due to greater access to health-promoting resources and less exposure to job insecurity (43). Low-income people usually go to the doctor only when their health problems become serious, instead of seeking preventive or early care (44, 45). This behavior may partly explain the observed inequality in healthcare utilization. Male-specific pattern is consistent with previous evidence from KSA showing that preventive healthcare use is concentrated among wealthier individuals, and with evidence from an international systematic review indicating that men’s healthcare-seeking behavior may be influenced by socioeconomic, cultural, and service-related factors (13, 46).

Within education groups, the highest income-related inequality in outpatient utilization was observed among individuals with primary education and intermediate education. This pattern may reflect variation in health coverage and access within these education strata, where many Saudis rely on GHC while non-Saudis typically depend on employer-sponsored PHI with benefit generosity that may vary by job type and income, potentially translating into differential access to providers and shorter waiting times. Consistent with Saudi evidence, lower education has been associated with lower use of services typically accessed through PHI pathways (13). Education may also be associated with preventive care use, as higher-educated individuals are more likely to utilize preventive services than less educated groups (13). Among individuals with higher education, utilization remained pro-rich, which may indicate that higher-income subgroups within this education level have better access to comprehensive employer-sponsored PHI and private providers, alongside higher utilization compared with their lower-income individuals (47). This finding is consistent with previous Saudi evidence showing that educational attainment is associated with preventive healthcare use, with higher-educated individuals more likely to use preventive services (13). It is also in line with decomposition evidence from Iran, suggesting that education, alongside income and insurance-related factors, can contribute to socioeconomic inequalities in healthcare utilization (48).

Pro-rich inequality in healthcare utilization was higher among unmarried individuals than among married individuals. Jung et al. showed that, in South Korea, married individuals, particularly those from higher-income households, are more likely to be covered by PHI and to use healthcare services compared with the unmarried (49). In a systematic review, marital status was associated with utilization of antenatal care, with married women generally more likely to attend healthcare visits than unmarried women (50). This pattern may also reflect the PHI guidelines, where married expatriates may upgrade PHI plans to cover spouses and children, which may be associated with fewer practical barriers to seeking care.

The results show that pro-rich inequality in healthcare utilization is greater among those without chronic conditions, which may be consistent with income-related differences in access to non-urgent, preventive, and early diagnostic services, which are more frequently used by wealthier individuals (51). In contrast, individuals with chronic diseases often require continuous interaction with the healthcare system and may therefore be more consistently engaged in formal healthcare services, regardless of their income level, which may be associated with lower observed inequality (15). In the Saudi context, this pattern may also reflect that governmental healthcare services are provided free of charge to citizens, while most expatriates entering the country for employment are generally free of chronic diseases due to mandatory pre-employment health screening, and their younger working age (38). This pattern is consistent with Saudi evidence showing that preventive health check-ups are concentrated among wealthier individuals, and with evidence from China suggesting that socioeconomic factors can influence healthcare utilization among patients with chronic conditions, although patterns may vary by service type and country context (13, 52).

The Northern region showed the highest level of inequality, whereas the Eastern region showed the lowest. These geographic disparities may be associated with the uneven distribution of primary health centers (PHCs), hospitals, and specialized clinics, particularly in remote and low-density areas such as the northern and southern borders, where service availability may be more limited (53). Although recent reforms under Vision 2030, including health clusters and digital health expansion, have aimed to improve healthcare delivery, the findings suggest that regional inequalities in healthcare utilization remain an important policy concern (54–56). These regional findings are in line with Saudi evidence showing regional differences in socioeconomic inequality in preventive healthcare use, and with evidence from a systematic review of studies in Global North countries suggesting that travel time and distance to healthcare services may influence geographic differences in healthcare utilization and outcomes (57, 58).

This study found significant pro-rich income-related inequality in outpatient healthcare utilization in KSA using data from the 2018 SFHS. The pro-rich distribution was more pronounced within the PHI group than in the GHC group and was particularly evident among non-Saudis and among participants with low education levels. In the decomposition analysis, Saudi nationality was the largest positive contributor to the observed inequality, whereas PHI itself made a small negative contribution, possibly due to its smaller share among the total sample.

### 4.1 Strengths and limitations

A key strength of this study is the use of a nationally representative dataset and the application of the Erreygers concentration index (ECI) with decomposition, which improves the generalizability of the findings and helps identify key contributors to observed income-related inequality in outpatient utilization. The use of the ECI is appropriate for a bounded (binary) utilization outcome, and the stratified analyses by health coverage type provide additional insight into inequality patterns within PHI and GHC groups.

The limitations include a cross-sectional design, which restricts causal inference, and reliance on self-reported utilization, which may underestimate some disparities. Outpatient utilization was measured as a binary indicator and, therefore, does not capture the frequency or intensity of visits. Moreover, the analysis was limited to data from 2018. However, the 2018 wave provides important pre-extension evidence before the full implementation of employer-mandated PHI for Saudi private-sector employees in 2019. Although the released SFHS analytical file was described by GASTAT as self-weighted, survey design identifiers such as strata and primary sampling units were not available, limiting our ability to account for the complex survey design in variance estimation. Monthly household income was available only in categories and was not equivalized (adjusted) for household size, which may introduce measurement error in the income ranking. Need was only partially captured using available proxies (age, perceived health status, and chronic disease) and may differ systematically between population groups (e.g., due to expatriate screening and healthy-worker effects). Therefore, these variables should be interpreted as available proxies for healthcare need rather than direct or comprehensive measures of need. In addition, the released SFHS dataset did not include disease-specific information on diabetes mellitus and hypertension, detailed information on facility type, direct measures of unmet need, or key access-related factors, such as benefit generosity, provider-network restrictions, and waiting times, which may also influence the observed income-related inequality. Consequently, the effects of income and differences in PHI coverage generosity could not be examined separately.

## 5 Conclusion

The explained component of inequality appeared to be more consistent with structural and enabling factors than with the available need-related proxies (self-rated health and presence of chronic disease). Although benefit generosity, provider-network restrictions, and unmet need were not directly measured, the findings highlight the importance of monitoring effective outpatient coverage and access conditions during Vision 2030 health-sector reforms. Efforts to reduce income-related inequality in outpatient care may require attention not only to coverage expansion, but also to variation in access conditions across population groups and regions.

These findings may support the development of equity-oriented health coverage policies that consider differences in healthcare utilization across income groups. During implementation, policymakers may consider incorporating equity indicators into routine evaluation of health coverage reforms to assess whether expanded coverage translates into more equitable outpatient healthcare utilization. This approach may help identify population groups or regions that require targeted policy measures and support progress toward universal health coverage in KSA.

## Data Availability

The data analyzed in this study is subject to the following licenses/restrictions: The 2018 Saudi Family Health Survey microdata are not publicly available due to privacy and confidentiality restrictions. Access to the dataset can be obtained only through the General Authority for Statistics (GASTAT), Kingdom of Saudi Arabia, subject to official permission. Requests to access these datasets should be directed to General Authority for Statistics (GASTAT), Kingdom of Saudi Arabia. Requests for access to microdata can be made through the GASTAT Microdata Request service: https://www.stats.gov.sa/en/request-for-scientific-use-files.
